# Effects of home-based, low-intensity exergaming on cognitive function of individuals with mild cognitive impairment: a study protocol for a randomized controlled trial

**DOI:** 10.1186/s12877-025-06054-w

**Published:** 2025-06-04

**Authors:** Sirintip Kumfu, Somporn Sungkarat, Sirinun Boripantakul, Piangkwan Sa-nguanmoo, Siriporn C. Chattipakorn

**Affiliations:** 1 Department of Physical Therapy, Faculty of Associated Medical Sciences, Chiang Mai University, Chiang Mai, Thailand; 2 Neurophysiology Unit, Cardiac Electrophysiology Research and Training Center, Faculty of Medicine, Chiang Mai University, Chiang Mai, Thailand; 3 Department of Oral Biology and Diagnostic Sciences, Faculty of Dentistry, Chiang Mai University, Chiang Mai, Thailand

**Keywords:** Low-intensity, Exergame, Mild cognitive impairment, Home-based exercise, Cognitive function

## Abstract

**Background:**

Although older adults with Mild Cognitive Impairment (MCI) face an increased risk of dementia, accumulating evidence has revealed that their cognitive functions could revert to normal levels with effective interventions. Exercise, particularly a combination of physical and cognitive training delivered in the form of an exergame, has shown promising effects in improving cognitive function of older adults with MCI. Nonetheless, previous research often prescribed center-based, moderate to vigorous intensity exercise, posing potential risks to older adults with physical or medical conditions as well as limiting accessibility for those with schedule or transportation constraints. Therefore, this study aims to evaluate the effects of home-based, low-intensity, combined physical-cognitive exercise in the form of an exergame, on cognitive function in individuals with MCI and to further investigate potential biomarkers linking the effectiveness of the exercise program to cognitive alterations.

**Methods:**

Sixty-four older adults with MCI will be enrolled and randomly allocated to either the exercise group or the control group. The exercise group will engage in a low-intensity, combined physical-cognitive exercise through an exergame, with a 50-minute session, 3 times per week for 12 consecutive weeks. The control group will not receive any intervention. Primary outcome measures will be cognitive performance (global cognition, executive function, memory, and attention), and secondary outcome measures will be plasma biomarkers and physical performance. All assessments will be administered at baseline and after a 12-week intervention.

**Discussion:**

The findings of this study might provide valuable insights into an effective and practical intervention program aimed at improving cognitive function of older adults with MCI. The low-intensity, home-based exergaming could have considerable clinical implications, as it has the potential to enhance accessibility for individuals who are unable to engage in high-intensity exercise or attend center-based exercise programs.

**Trial registration:**

ClinicalTrials.gov identifier: NCT06201533, Registered January 11, 2024.

## Background

With the world’s aging population, dementia, particularly Alzheimer’s disease (AD) has become a global pandemic. It is estimated that the number of people with dementia worldwide will reach 74.7 million in 2030 and 139 million in 2050 [1, 2]. Dementia is preceded by a preclinical phase, a syndrome called Mild Cognitive Impairment (MCI). Older adults with MCI are approximately 10 times more likely to develop dementia compared to cognitively intact older adults [3]. However, MCI is a potentially reversible condition, several individuals with MCI could revert to normal levels of cognition [4]. Therefore, interventions that could prevent cognitive deterioration and/or improve cognitive function in this population would significantly decrease the incidence of dementia.

Non-pharmacological intervention particularly exercise has shown promise in MCI management. Accumulating evidence has demonstrated that exercise could enhance cognitive function, reduce the risk of dementia, and improve physical performance and general quality of life in individuals with cognitive impairment [5–8]. The beneficial effects of exercise on cognition have been demonstrated for both global cognitive function and specific cognitive domains particularly memory, attention, and executive function in individuals with MCI [9, 10]. Among several types of exercise, a systematic review and meta-analysis revealed that the most profound effects are observed in combined physical-cognitive exercise [6]. Previous research has shown that individuals with MCI demonstrate greater improvement in cognitive function (e.g., learning and memory), hippocampal BDNF, and physical functioning when engaging in combined physical-cognitive training compared to either cognitive or physical training alone [11, 12]. The explanation for this phenomenon is that as each intervention is associated with different mechanisms, the neuroplasticity and synaptic integrity are boosted when the two interventions are combined [13].

With current technologies, simultaneous physical and cognitive exercise can be delivered in the form of an exergame, an exercise that involves moving the body to interact with the game. Exergames specifically designed for older adults have been proven to be safe, easy to use, and enjoyable, which in turn results in a high adherence rate [14, 15]. Exercise adherence has an important contribution to the success of an intervention. The effectiveness of exercise depends not only on the exercise program itself but also on the individual’s engagement. The meta-analysis study findings suggest that improvements in cognitive function were greater in samples where better adherence to the exercise training was reported, for both participants with AD and those at risk of AD [16]. Several studies demonstrated that exergames improve physical functions (e.g., balance, mobility, physical fitness, and gait) and cognitive function (e.g., executive functions, memory, and processing speed) among healthy older adults, older adults with metabolic syndrome, MCI, and dementia [14, 15, 17, 18].

Recently, research has expanded beyond investigating the impact of exercise on cognitive function to determining the underlying mechanisms by which exercise affects cognitive function in individuals with MCI. One of the most consistently observed effects of exercise is an increase in the level of plasma brain-derived neurotrophic factor (BDNF). Previous research suggested that exercise enhances cognitive performance, likely through the upregulation of BDNF in circulation [5, 19–22]. Additionally, several studies have examined the influence of moderate to vigorous exercise on inflammatory biomarkers, especially pro-inflammatory cytokines such as interleukin 6 (IL-6), interleukin 1β (IL-1β), tumor necrosis factor-alpha (TNF-α), and C-reactive protein (CRP) [5, 23]. For example, a previous study found that engaging in physical exercise enhances cognitive performance and BDNF levels while downregulating IL-6 levels [20]. Recently, there has been a growing interest in investigating the effects of exercise on irisin and fibroblast growth factor 21 (FGF21). Emerging evidence has shown that irisin plays a role in enhancing synaptic functions in the brain [24]. Since approximately 72% of irisin is produced from muscle secretion, exercise may potentially be an effective method to stimulate the release of irisin, which may in turn improve cognitive function [25]. Previous clinical research demonstrated that a high level of FGF21 was independently associated with cognitive impairment in patients with metabolic syndrome [26], however, exercise interventions could reduce FGF21 levels in obese women and elderly men [27, 28]. Nevertheless, the effects of exercise training on FGF21 levels in individuals with MCI have never been investigated.

Apart from types of exercise, intensity also plays a vital role in the effectiveness of exercise. Previous studies mainly focused on moderate to vigorous exercise. While the promising effects on cognition are evident, some older adults may have limitations or contraindications, such as arthritis, hypertension, and heart disease, which preclude their participation in moderate and high-intensity exercises. Recently, there has been a growing interest in investigating the effects of low-intensity exercise on cognitive function. A limited number of studies found that low-intensity exercise could improve memory [29] and other cognitive functions [30–34] in adults and older adults without cognitive impairment. However, it is unknown whether low-intensity exercise would be beneficial for individuals with MCI. Other common barriers to engaging in regular exercise include a lack of time, schedule conflicts, and transportation constraints, as most exercise training programs are center-based. With those barriers, home-based exercise appears to be a viable alternative. Home-based exercises have been demonstrated to reduce the rate of cognitive decline [35], as well as improve global and specific cognitive function in older adults with and without MCI [36, 37]. Collectively, this study aims to evaluate the effects of home-based, low-intensity, combined physical-cognitive exercise (in the form of an exergame) on cognitive function in individuals with MCI and explores plasma biomarkers potentially underpinning these effects.

## Methods

### Trial design

**Fig. 1:**
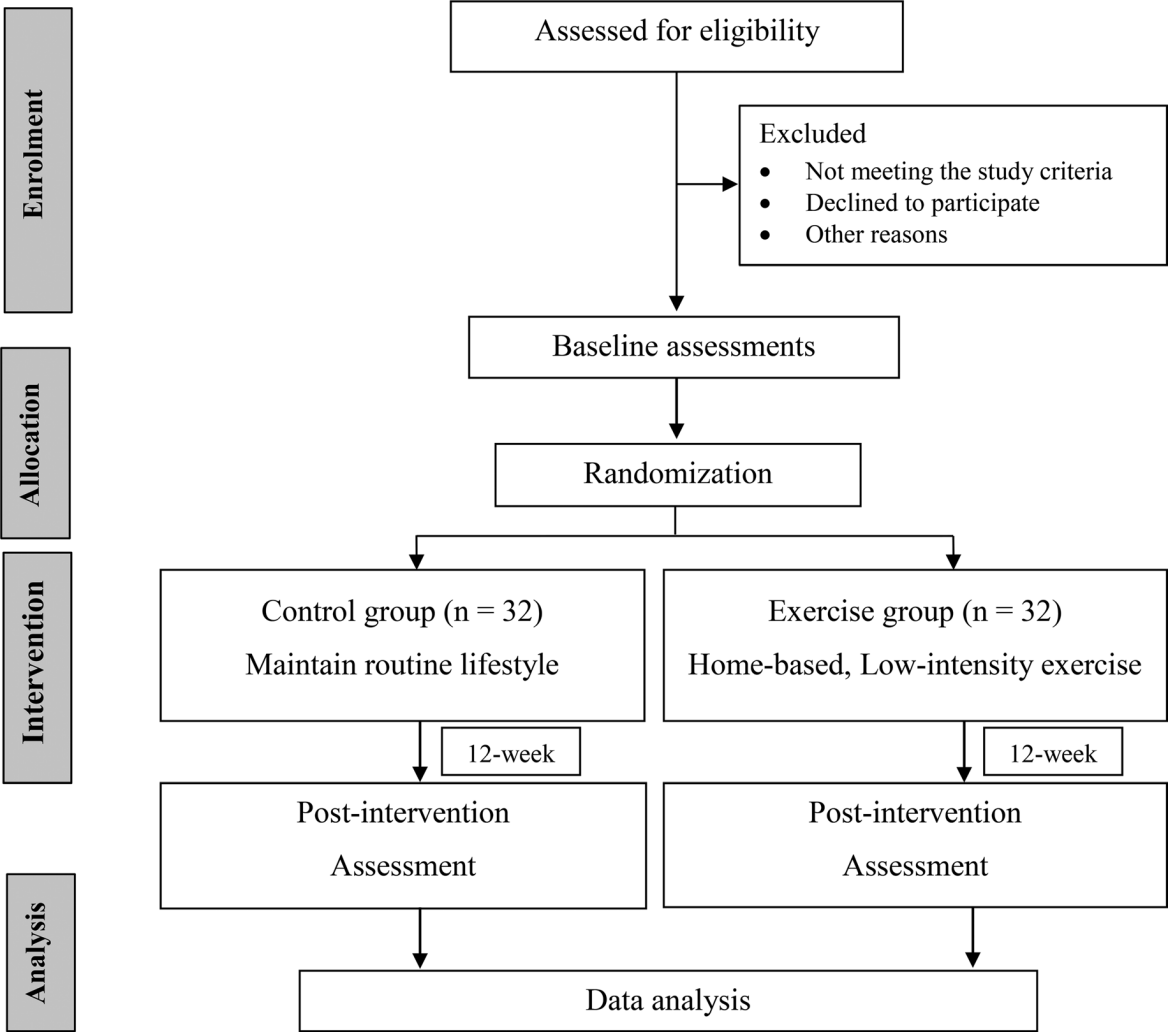
Flow diagram of the study procedure

This is a randomized controlled trial (RCT) with two parallel groups and single-blind outcome assessors. The study protocol conforms to the Consolidated Standards of Reporting Trials (CONSORT) guidelines in which the flow diagram is shown in Fig. 1.

### Study setting

The intervention will take place in a home-based setting. Baseline and post-intervention assessments will be conducted at the Faculty of Associated Medical Sciences, while blood biomarkers will be collected at the Prompt Health Center, Faculty of Associated Medical Sciences, and analyzed at the molecular biology unit of the Cardiac Electrophysiology Research and Training Center, Faculty of Medicine, Chiang Mai University.

### Participants

Participants will be community-dwelling older adults aged 60 years or older. To be eligible for the study participants have to meet the criteria for MCI based on the recent DSM-V criteria [38], comprehend instructions, have adequate vision and hearing, and have physical performance sufficient to exercise at home safely as determined by a score more than 9 of 12 points on short physical performance battery (SPPB) [39]. Exclusion criteria included dementia as determined by a score less than 22 of 30 points on mental state examination T10 (MSET10) [40], depressive symptoms as determined by a score more than 6 of 15 points on Thai geriatric depression scale (TGDS) [41], being diagnosed with other neurological conditions that affect cognition and mobility (e.g., Parkinson’s disease, Stroke, Multiple Sclerosis, and AD), acute or/and chronic disease that could not be controlled (e.g., Arthritis, Asthma, Hypertension, Diabetes mellitus, and Coronary Artery Disease), and exercise regularly (40–50 min/day at least 3 days/week) during the past 3 months [42].

### Recruitment

Participants will be recruited from communities through the distribution of flyers, engagement with community leaders, and utilization of social media. The recruitment process will employ a multimethod approach, incorporating both online and offline advertising targeted towards local communities, health organizations, and companies.

### Randomization and allocation concealment

Participants meeting the eligibility criteria will be randomly allocated to the exercise or control group. Participants will be stratified by age (60–65, 66–69, 70–75, 76–80, or > 80 years), gender (male or female), education level (≤ 12 or > 12 years), and co-morbidity (1–2, or > 2 types) and randomized using a permuted block randomization with varying block size [4, 6, 8] and 1:1 allocation. Randomization will be administered by an individual external to the study using a computer-generated list and concealed within securely sealed, opaque envelopes. All outcome measures will be performed by trained assessors who are blinded to the participant’s group allocation. The researcher will be blinded to the outcome assessments.

### Interventions

#### Exercise group

A low-intensity, combined physical-cognitive exercise will be delivered in the form of an exergame, where the participants move their bodies to interact with the game. The cognitive training, which focuses on memory, attention, and executive function (e.g., anticipation, planning, switching, and inhibition), along with the physical exercise, which focuses on aerobic and balance training, will be incorporated into the game (Table 1). There will be three games with three levels of difficulty (beginner, intermediate, and advanced). Training progression will depend on the participant’s performance. To progress to the next level, participants must achieve a score exceeding 50% at their current level. Prior to training, there will be a familiarization phase to ensure that each participant can safely perform the exercises at home and that the intensity is at a low level. In the training phase, the participants will engage in a 50-minute exercise (7 min warm-up, 36 min of exercise, and 7 min cool-down), 3 times per week for 12 consecutive weeks.

In the familiar phase of exercise, all participants will wear a heart rate monitor watch. The watch will be set at low-intensity ranging between 30 and 39% of each participant’s heart rate reserve (HRR). The Karvonen formula will be used to calculate the HRR (estimated maximal HR − resting HR) and the target HR during exercise (HRR x percentage of training intensity + resting HR) [43]. In addition, they will be subjectively monitored using Borg’s Rating of Perceived Exertion (RPE) scale, with a range of 9–11 out of 20.

**Table 1:**
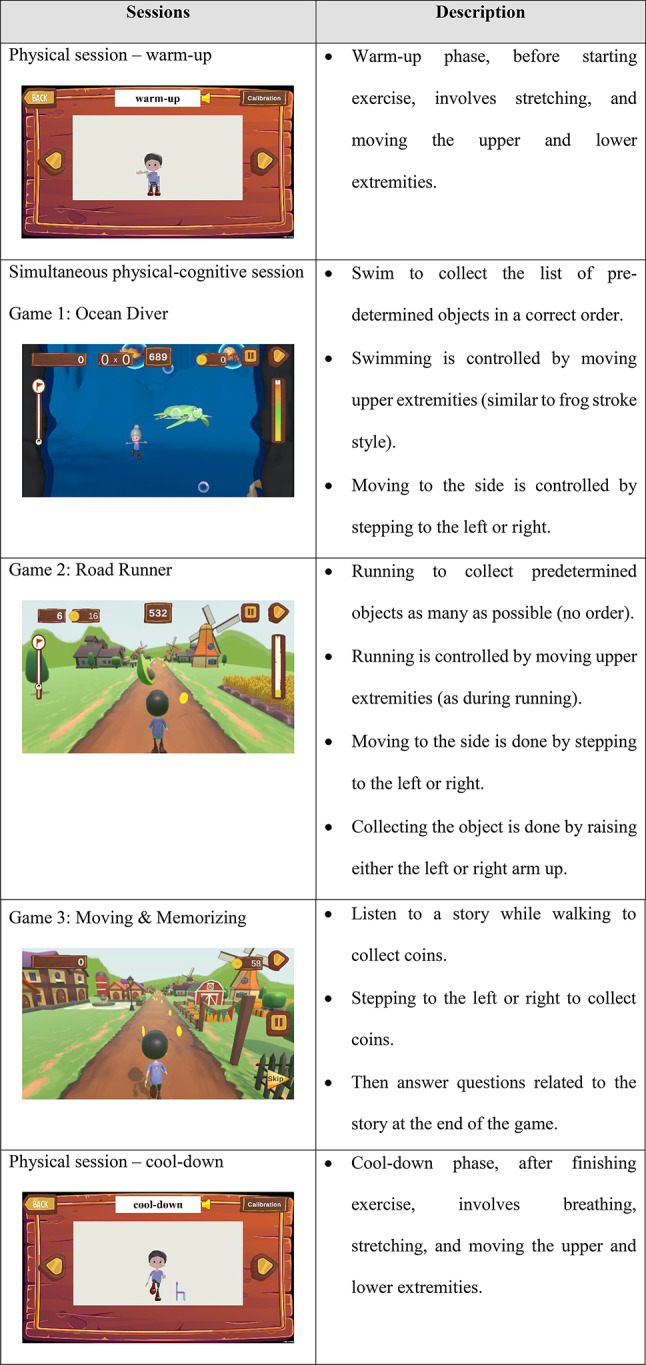
Summary of the characteristics of the exercise program

#### Control group

Participants in the control group will not receive any exercise or intervention and be asked to maintain their routine lifestyle. They will be offered the same program as the exercise group after finishing the study.

### Adherence

Exercise adherence will be monitored through the participant’s exercise diary and a record in the exergame program. They will also receive a weekly phone call from the research team to address any questions about the exercise and to provide exercise reminders.

### Adverse event

Participants will be instructed to immediately report any adverse events occurring during the study. Exercise termination will follow the American College of Sports Medicine (ACSM) standard guideline (e.g., moderate to severe chest pain, severe or unusual shortness of breath, etc.) [44].

### Assessment and outcome measures

Demographic data of the participants which consisted of age, gender, body mass index (BMI), education level, medical conditions, diet, supplements, lipid profile (high-density lipoprotein cholesterol (HDL), total cholesterol, low-density lipoprotein cholesterol (LDL)), glucose level (Fasting Blood Sugar-FBS) and history of falls in the past year will be obtained. Primary outcome measures will be cognitive performance. Secondary outcomes will be plasma biomarkers via ELISA assays (including BDNF, IL-6, irisin, and FGF21) and physical performance. All assessments will be administered at baseline and post 12-week intervention (Table 2).

**Table 2:** Schedule of enrolment, interventions, and measurements for outcome measures

	Study period			
	**Enrollment**	**Allocation**	Post allocation	**Post-intervention**
**Timepoint**	-T1	T0	0–12 weeks	T1 (12 weeks)
**Enrollment**:				
Eligibility screen	x			
Informed consent	x			
Allocation		x		
**Interventions**:				
Exercise group			↔	
Control group			↔	
**Measurement**:				
ADAS-Cog		x		x
LMT		x		x
TMT		x		x
BDNF		x		x
IL-6		x		x
Irisin		x		x
FGF21		x		x
TUG		x		x
30-s CST		x		x

### Primary outcomes

Primary outcome measures will be cognitive function (global cognition, executive function, memory, and attention). Alzheimer’s Disease Assessment-cognitive subscale (ADAS-Cog) has been verified to be useful in screening cognitive function among individuals with MCI [45]. ADAS-Cog will be used to evaluate the participant’s global cognitive function. The ADAS-Cog assesses performance on 11 cognitive tasks: orientation, a 10-word list learning task, a 12-word recognition task, recall of instructions, comprehension of commands, object and finger naming, word-finding difficulty, expressive language, language comprehension, ideational praxis, and constructional praxis. Individual items and the total score will be recorded. Logical memory test (LMT), a subtest of the Wechsler Memory Scale will be used to evaluate episodic memory. Participants will be asked to listen carefully to 2 short stories and remember their content. After a 30-minute delay, they will be asked to repeat each story as accurately as possible. A score is assigned based on the number of correct details recalled. The Trail Making Test (TMT) will be used to assess attention (part A) and executive function (part B-A). In TMT part A, participants will be instructed to draw a line to connect consecutive numbers in numerical order (e.g., 1-2-3) as quickly and correctly as possible. For TMT part B, participants will be asked to draw a line to connect consecutive numbers in numerical order and letters in alphabetical order in an alternating sequence as quickly and correctly as possible. The time taken to complete TMT part A will be used to evaluate attention and the time difference between parts B and A will be used to assess executive function.

### Secondary outcomes

Secondary outcome measures will be plasma biomarkers (BDNF, IL-6, irisin, and FGF21) and physical performance (Timed Up and Go test and 30-second sit to stand test). As for blood collection protocol: twelve-hour fasting blood samples will be collected for 15 ml between 8.00 AM and 9.00 AM and kept in tubes that contain anticoagulants. Blood sampling will be rescheduled if participants report illness or use of anti-inflammatory agents. The participants will be instructed to refrain from exercise, and alcohol for 24 h before each visit. Plasma will be obtained by centrifugation of the blood for 10 min and kept at -80 °C immediately until analysis. Commercially available quantitative sandwich enzyme-linked immunosorbent assay kits will be used for the analysis of BDNF, IL-6, irisin, and FGF21, following the protocols provided by the test manufacturer.

Physical performance assessment will be Timed Up and Go (TUG) single and dual-task and 30-second sit to stand test (30-s CST). For TUG, participants will be instructed to rise from a standard-height chair, walk forward 3 m at their fastest speed, turn 180 degrees at the mark, walk back to the chair, and sit down [46]. The time (in sec) taken to complete the task will be recorded. They will perform: the TUG (single TUG) and the TUG in concurrent with counting backward by three (dual TUG). As for the 30-second sit to stand test, participants will be instructed to sit in the middle of the chair with their back straight, feet flat on the floor, and arms crossed at the wrists, held against the chest. They will be requested to stand up and sit down in the chair repeatedly as many times as possible for 30 s [47]. The total number of sit-to-stands completed within 30 s will be recorded.

### Sample size calculation

The sample size for the study was determined for the primary outcome measures based on previous studies that examined the effects of Tai Chi which could be considered as combined physical-cognitive exercise at low-to-moderate intensity [48, 49]. The previous findings on memory and executive function yielded effect sizes of 0.40 and 0.38, respectively [49]. Thus, the sample size was estimated using the lowest effect size (i.e., executive function). With 80% power and 5% type I error, a total sample size of 57 (29 per group) will be required to detect change. To accommodate 10% dropout, a total of 64 participants (32 per group) will be enrolled in the study.

### Statistical analysis

Data normality will be performed using the Shapiro-Wilk test. Descriptive statistics will be used to describe the demographic characteristics of the participants. If data are normally distributed, the independent samples t-test (for continuous data) and chi-square test (for categorical data) will be conducted to determine differences in demographic data between the intervention and control groups at baseline. A two-way mixed-model ANOVA will be used to compare the outcome measures across the two different assessment intervals (at baseline and the end of 12 weeks) and between the two groups (exercise and control groups). Bonferroni will be used for the post-hoc analysis in this study. However, if data are not normally distributed, non-parametric statistics will be performed. Specifically, the Mann-Whitney U test will be used for pairwise comparisons between groups, and the Wilcoxon signed-rank test will be used to compare the outcome measures between baseline and the end of 12 weeks. Data in this study will be analyzed using the intention-to-treat (ITT) approach. Missing data will be handled using multiple imputations. The alpha level will be set at 5%.

### Data management

Each participant will be assigned a unique identifier code. The study data will be securely stored with restricted access, adhering to institutional ethics committee guidelines. All information will be maintained in a secure storage facility for three years post-study completion.

## Discussion

The primary aim of this study is to examine the effects of home-based, low-intensity, combined physical-cognitive exercise on cognitive function of individuals with MCI. To the best of our knowledge, there have not been any intervention trials assessing the impact of home-based, low-intensity exergame on cognitive function in this population. It is anticipated that the game-based exercises will provide enjoyment, while the home-based setting will help overcome common barriers such as schedule conflicts, transportation issues, and family responsibilities, thereby enhancing training adherence. Additionally, plasma biomarkers potentially linking the low-intensity, combined physical-cognitive training to cognitive changes will be explored. The findings of this study might offer an effective intervention program to improve the cognitive and physical performance of older adults with MCI. This program might have important clinical implications as its low-intensity and home-based format would extend accessibility to individuals with MCI who are unable to engage in high-intensity exercise or attend center-based exercise programs. Further, the knowledge gained from the results of the plasma biomarkers would provide a better understanding of the mechanisms underpinning the therapeutic effects of low-intensity, combined physical-cognitive exercise on cognitive function which will have a significant impact on the prevention and management of cognitive impairment.

There are some limitations to this study. First, while the use of a home-based, technology-driven exergame intervention may enhance enjoyment and adherence, it may reduce participation among older adults with low computer or digital literacy. Second, variability in home environments may influence participants’ ability to engage with and benefit from the intervention. To minimize these limitations, a home visit will be conducted prior to the beginning of the home-based intervention to perform an initial home assessment and assist participants with the set up and operation of the exergames. Step-by-step user instructions will be provided for reference. Based on the home assessment, recommendations will be provided to optimize the home setting, promoting safety and a standardized exercise setup. During the home visit, family members or caretakers will be invited to participate, so they can assist the participants throughout the course of the home-based exercise program.

## Data Availability

No datasets were generated or analysed during the current study.

## Declarations

### Dissemination

The study will be disseminated through publication in peer-reviewed journals, as well as presentations at relevant conferences.

## Abbreviations

AD: Alzheimer’s disease
ADAS-Cog: Alzheimer’s Disease Assessment-cognitive subscale
BDNF: Brain-Derived Neurotrophic Factor
FGF21: Fibroblast Growth Factor 21
IL: Interleukin
LMT: Logical Memory Test
MSET10: Mental State Examination T10
MCI: Mild Cognitive Impairment
TGDS: Thai Geriatric Depression Scale
TMT: The Trail Making Test
TUG: Timed Up and Go
