# Correction: Effects of home-based, low-intensity exergaming on cognitive function of individuals with mild cognitive impairment: a study protocol for a randomized controlled trial

**DOI:** 10.1186/s12877-025-06921-6

**Published:** 2026-01-19

**Authors:** Sirintip Kumfu, Somporn Sungkarat, Sirinun Boripantakul, Piangkwan Sa-nguanmoo, Siriporn C. Chattipakorn

**Affiliations:** 1 Department of Physical Therapy, Faculty of Associated Medical Sciences, Chiang Mai University, Chiang Mai, Thailand; 2 Neurophysiology Unit, Cardiac Electrophysiology Research and Training Center, Faculty of Medicine, Chiang Mai University, Chiang Mai, Thailand; 3 Department of Oral Biology and Diagnostic Sciences, Faculty of Dentistry, Chiang Mai University, Chiang Mai, Thailand

**Correction:** **BMC Geriatr 25, 408 (2025)**

10.1186/s12877-025-06054-w

Following publication of the original article [1], the authors reported that the ethical approval number in the ‘Ethics approval and consent to participate’ section was incorrect. Below is the correct statement.

## Notes

**Incorrect:**

## Notes

**Ethics approval and consent to participate**

## Notes

The research protocol has been approved by the Human Ethical Review Board of the Faculty of Associated Medical Sciences, Chiang Mai University, Thailand (approval number: AMSEC-63EX-102). All participants will provide written informed consent prior to participation.

## Notes

**Correct:**

## Notes

**Ethics approval and consent to participate**

## Notes

The research protocol has been approved by the Human Ethical Review Board of the Faculty of Associated Medical Sciences, Chiang Mai University, Thailand (approval number: AMSEC-67EX-005). All participants will provide written informed consent prior to participation.
